# Biology, Ecology and Management of Tephritid Fruit Flies in China: A Review

**DOI:** 10.3390/insects14020196

**Published:** 2023-02-16

**Authors:** Yuxin He, Yijuan Xu, Xiao Chen

**Affiliations:** 1 Guangdong Laboratory for Lingnan Modern Agriculture, Department of Entomology, South China Agricultural University, Guangzhou 510642, China; heyuxin@stu.scau.edu.cn; 2 Henry Fok School of Biology and Agriculture, Shaoguan University, Shaoguan 512005, China

**Keywords:** tephritid, biological parameters, ecological performance, integrated pest management

## Abstract

**Simple Summary:**

Tephritid fruit flies are widely distributed around the world and lay eggs in fruits and vegetables, resulting in rotting and economic losses. To limit economic loss caused by these flies, we reviewed and summarized three decades of literature on 10 important fly species occurring in China. We summarized the biology, ecology and integrated control methods to help researchers, quarantine officials and even hobbyists obtain more basic knowledge and a more innovative outlook.

**Abstract:**

Tephritid fruit flies are notoriously known for causing immense economic losses due to their infestation of many types of commercial fruits and vegetables in China. These flies are expanding, causing serious damage, and we summarized references from the last three decades regarding biological parameters, ecological performance and integrated pest management. There are 10 species of tephritid fruit flies mentioned at a relatively high frequency in China, and a detailed description and discussion in this comprehensive review were provided through contrast and condensation, including economics, distribution, identification, hosts, damage, life history, oviposition preference, interspecific competition and integrated management, in anticipation of providing effective strategies or bases for the subsequent development of new research areas and improvement of integrated management systems.

## 1. Introduction

Tephritid fruit flies belong to a large group of insects in Diptera: Tephritidae and are widely distributed around the world, with more than 500 genera and 5000 species [1]. Among them, harmful species are mainly distributed in six genera, *Anastrepha*, *Bactrocera*, *Ceratitis*, *Zeugodacus* and *Rhagoletis* [2,3]. These flies are notorious for causing immense economic losses due to their infestation of many types of commercial fruits and vegetables; therefore, significant effort is carried out in the risk analysis progress, and takes substantial investments for control [4]. Moreover, these flies are highly valued quarantine or invasive agricultural pests internationally that have the characteristics of rapid spread, invasiveness and destructiveness [5,6].

This paper presents an extensive collection of literature based on the Chinese context to provide useful local knowledge for researchers, quarantine officials, and industry biosecurity experts on tephritid fruit flies. In addition to being searchable in PubMed and Web of Science (WOS), the majority of Chinese studies on these flies are included in the China National Knowledge Infrastructure (CNKI), Wanfang Database, and China Science and Technology Journal Database, for which we have compiled and summarized nearly three decades of information that targeted the large and dangerous pest group, such as the genera *Bactorcera*, *Carpomya*, *Rhagoletis* and *Zeugodacus,* which are the most studied in China, especially *Bactrocera correcta* (Bezzi), *Bactrocera dorsalis* (Hendel), *Bactrocera latifrons* (Hendel), *Bactrocera minax* (Enderlein), *Bactrocera tsuneonis* (Miyake), *Carpomya vesuviana* (Costa), *Rhagoletis batava obseuriosa* (Kolomiets), *Zeugodacus cucurbitae* (Coquillett), *Zeugodacus scutellatus* (Hendel) and *Zeugodacus tau* (Walker). Focusing on the perspective of biosafety risk analysis and assessment, we summarize and provide an outlook on the characteristics of the biology, ecology and integrated management of these flies in China, including economic importance, distribution, species identification, host range and damage characteristics, life history, oviposition and host preference, and interspecific competition and details and examples of control methods, which provide effective strategies or bases for the subsequent development of new research areas and the improvement of integrated management systems.

## 2. Economic Importance and Distribution

### 2.1. Economic Importance

Tephritid fruit flies cause damage not only to hosts but also to the development of related industries and economic income. Because of the most serious damage and the greatest number of host species, *B. dorsalis* and *Z. cucurbitae* have always been used as examples. By constructing an index system for assessing the economic loss caused by *B. dorsalis*, the total economic loss to Fujian Province in 2005 was calculated as 14.9 million dollars, of which the direct economic loss was 13.4 million dollars and the indirect economic loss was 1.5 million dollars [7]. Using the @RISK model to predict the direct economic loss caused by *Z. cucurbitae* in China, under the scenarios of no control and control, the annual direct economic losses were 666.6–3551.5 million dollars and 202.3–2141.8 million dollars, respectively, and the annual direct economic loss that could be recovered under control was 337.7–1613.9 million dollars [8]. In addition to the economic loss, in agricultural areas, the cost of prevention and management is also part of the economic expenditure. For Hainan melon households, the yield of bitter melon is 1500 kg/667 m^2^ in winter, and the selling price is 0.98 dollar/kg, so households could receive 1465.2 dollar/667 m^2^ in gross profit; based on an investigation of the same area, Hainan melon households need to spend 81.4 dollars on management costs (only 5.5% of gross profit, but including pesticides, labor and machinery depreciation), with a prevention and control effect of 85% [9].

### 2.2. Distribution

**Figure 1:**
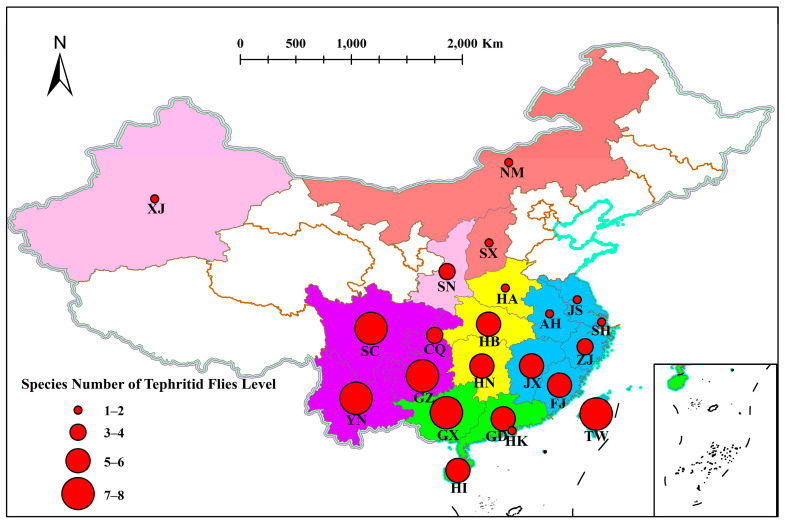
Distribution of tephritid fruit flies in each province of China. The size of the solid circles in the figure represents the species number of tephritid fruit flies. Colors in the figure represent different regions (Red, yellow, green, blue, purple, and pink represent North, Central, South, East, Southwest, and Northwest China respectively). Abbreviations: AH, Anhui; CQ, Chongqing Shi; FJ, Fujian; GD, Guangdong; GX, Guangxi Zhuangzu Zizhiqu; GZ, Guizhou; HA, Henan; HB, Hubei; HI, Hainan; HK, Hong Kong; HN, Hunan; JS, Jiangsu; JX Jiangxi; NM, Nei Mongol Zizhiqu; SC, Sichuan; SH, Shanghai Shi; SN, Shaanxi, SX, Shanxi; XJ Xinjiang Uygur Zizhiqu, TW, Taiwan; YN, Yunnan; ZJ, Zhejiang. The map data was generated by Geospatial Data Cloud (https://www.gscloud.cn, accessed on 4 February 2022) and Alibaba Cloud (DataV.GeoAtlas, http://datav.aliyun.com/portal/school/atlas/area_selector, accessed on 4 February 2022). The spatial analysis function was via ArcGIS (version 10.7) and Mapshaper (https://mapshaper.org, accessed on 4 February 2022).

The data presented in Figure 1 and Table 1 were obtained from literature reports with the field evidence. Fujian and Taiwan Provinces in East China, Guangdong Province and the Guangxi Zhuang Zizhiqu in South China, and Guizhou, Sichuan and Yunnan Provinces in Southwest China are the areas where the tephritid fruit flies overlap many times. In subsequent studies, we observed the phenological changes in these overlapping areas and explain the mechanisms related to the introduction, occurrence and invasion of fruit flies in depth at the ecological level.

## 3. Morphological Characteristics

### 3.1. Basic Taxonomy

Tephritid flies not only cause direct economic damage to fruits and vegetables, but also increase huge costs for relevant quarantine or control programs because of the uncorrected morphology and identification [3]. In the process of literature review, we could also find that the taxonomy of Tephritidae has been constantly revised; especially in the adult stage, their markings and colors are very similar, which may be considered as under selective pressure or resulting from wasp mimicry [90], but they also have typical characteristics, such as *B. dorsalis* has a dark black and yellow coloration on the whole body, and most of the thorax back is black with obvious yellow “U”-shaped markings, while *Z. cucurbitae* has an obvious “T” shape [76,91].

But for the immature stage, the morphological differences are very subtle, so in the guidance of daily agricultural activities, that is, the popular science of farmers or primary-level agricultural departments, we generally only give a general description. The eggs are slightly curved and shuttle-shaped, one side is tapered, and the other side is oval, with a milky color in the newly hatched period that slowly transforms into a light-yellow color. The larvae are creamy white, with a pointed head, black mouth hook and thick tail, and go through three developmental stages, gradually increasing in length as the age increases. The pupae are oval-shaped, being light yellow initially and changing to reddish brown. There is a protrusion of valve remnants on the anterior end of the pupal body, with a slight constriction at the posterior end of the valve.

### 3.2. Molecular Identification

It is easy to confuse the morphological identification of tephritid flies, which produces erroneous results; in particular, the distinction between similar species of flies requires complete morphological structures and extensive practical experience and professional skills, while the immature stage is more difficult to identify because of extremely similar immature stages [92]. Molecular identification methods can greatly reduce the cost required for rearing to the adult state, and the application of molecular techniques can ensure the accuracy and objectivity of the identification results. These methods include isozymes, PCR, RAPD, RFLP, AFLP and SSR [92]. A technical system was established for the molecular identification of Chinese quarantine tephritids based on DNA barcoding technology, conventional PCR, quantitative real-time PCR and integrated flow path microarray technology [93]. Then, a DNA barcoding library of Chinese quarantine tephritids was constructed, mainly involving 27 species, and it was also possible to identify some species of tephritids in the nonadult state. Critically, DNA barcoding enables the accurate identification of target fly species other than the complex [93].

## 4. Host Range and Damage Characteristics

### 4.1. Host Range

Basically, the hosts listed in Table 2 were obtained from field surveys; the most serious damage was caused by the genus *Bactrocera*, especially *B. correcta* and *B. dorsalis*. The host range spanned a large area; among the hosts, the most infested fruits were concentrated in the Rutaceae and Rosaceae families, while vegetables were concentrated in the Cucurbitaceae and Solanaceae families.

### 4.2. Damage Characteristics

Tephritid fruit flies oviposit in the host plants, the hatching larvae directly feeding, and cause rotting and premature yellowing and loss of products [107,108]. It has also been shown that episodic enteritis leading to abdominal pain and diarrhea occurred in humans after the consumption of pernicious guavas, and maggots were then detected in the patient’s feces and identified by rearing as *B. dorsalis* [109].

## 5. Life History

Tephritid fruit flies have one or more generations per year [59]. Most are phytophagous taxa [110], and eclosion adults develop to sexual maturity during 6–13 days [111,112,113]. After sexual maturity, the female lays eggs under the skin of fruits, and the larvae are mature enough to pupate in the soil of hosts, usually overwintering as pupae, and then enter the next generation after the new adult eclosion. The specific developmental stages are as follows:

Egg hatching in the field varies seasonally, from approximately 1 day in summer, 2–3 days in spring and autumn, to 7–20 days in winter [114]. The developmental period of larvae varies from 9 to 23 days; if the absence of food or deterioration of food occurs, the mortality of 1st and 2nd instar larvae increases, whereas 3rd instar larvae may pupate earlier, or the developmental stages become longer, or body size is smaller [115].

The developmental period of the pupa becomes shorter with increasing temperature. Within a certain temperature range, the period of pupa lasts from 5 to 33 days [116], the prepupae transfer to pupate from 1 to 2 days [117], and the moisture content of the sand or soil in which the mature larvae enter affects the depth of pupation and the survival rate of the pupae [114]. There are also cases of diapause, such as the “double-edged” effect of low temperatures, which can induce diapause in *B. minax*; however, natural low temperatures in the field can also promote pupation and eclosion, but the release of diapause is not affected by the photoperiod [118,119].

Adults can eclose throughout the whole day but are most vigorous at 6 to 10 a.m. Newly eclosed adults crawl to a shady place to rest for 2 to 3 h and gradually begin to feed after their wings unfold [111,114,115,120]. Sexual maturation of posteclosion adults occurs after supplementation [111,121], and this process is followed by mating for approximately 8 to 15 days in daylight, especially in the early morning [115,122,123].

The flight ability of tephritids, especially *B. dorsalis*, does not decrease with sexual organ maturity but is strongest during the peak of oviposition. The maximum flight age is different from that of typical migratory insects, indicating that the phenomenon of “oogenesis conjugated with flight”, which is characteristic of migratory insects, does not exist in *B. dorsalis* [124]. Therefore, under natural conditions in the wild, tephritid fruit flies are capable of long-distance dispersal under certain conditions [125], and their flight ability is an important reason for their expanding distribution and occurrence area, as well as their reoccurrence after eradication [126,127].

## 6. Oviposition and Host Preference

When tephritid fruit flies oviposit, mated females will select certain species and varieties of hosts, and there may be differences in selectivity for the same host variety [128]. For example, *B. dorsalis* preferred local mangoes over other introduced species [18]. In addition, the damage caused by *B. minax* adult oviposition in three orange varieties was significantly different, and the damage rates of “Luo Qi”, “Wenzhou Tangerine” and “Za Gan” were 20.7%, 26.3% and 40.7%, respectively [129]. The selectivity of *Z. cucurbitae* for different varieties of mangoes was ranked as “3-year-old mango” > “Tainung No. 1” > “Tiger Leopard Tooth” > “Carmine mango” > “Hawksbill mango” [45].

Host color, maturity and size attributes also influence oviposition selection, with most tephritid fruit flies showing a strong tendency toward yellow [128]. For example, *B. dorsalis* clearly prefers the color orange, which is similar to the color that is close to that at maturity, and is more sensitive to orange, green and yellow colors in the wavelength range of 500 to 640 nm [130]. However, there are also other examples: *B. dorsalis* prefers black to yellow [107], but red was more effective in attracting *B. correcta* than yellow [131], and *Z. cucurbitae* was most phototropic to purple and white, followed by yellow [116]. The extent of damage caused by tephritid fruit flies increases as the fruit matures [128]. For instance, *B. dorsalis* had a bias in the selection of host maturity, with the damage rate of late-ripening varieties being higher than that of early-ripening varieties [18]. The most obvious physical manifestation of the difference between ripe and immature varieties is “hardness”; hence, *B. correcta* showed oviposition selection on the same species of fruits with different hardness, i.e., low hardness (overripe) > medium hardness (just ripe) > high hardness (unripe) [13].

The different tissue sites of the hosts and whether they are injured also play a role in oviposition selection [128]; for example, *B. dorsalis* preferred to oviposit on banana flesh rather than the peel, but preferred mango peel to the flesh, and the number of eggs oviposited at different tissue sites reached a significant level of difference [132]. In addition, there was high selectivity for artificially damaged pomegranates on which the highest number of eggs was laid. In addition to the factors described above, the interactions between multiple factors need to be considered [133].

## 7. Interspecific Competition

Interspecific competition is a common phenomenon among insects, especially among closely related species, and is often accompanied by invasion phenomena. The invasion of tephritids is systematically accompanied by a large change in the number or extinction of local/foreign colonies, and this invasion-induced change in the interspecific population can be attributed to competitive interspecific substitution or exclusion [134,135]. An in-depth understanding of interspecific competition in tephritids can theoretically explain the intrinsic mechanisms and verify the hierarchical pattern, as well as provide biological evaluation indicators for predicting the potential and success of invasive tephritid fruit flies [136].

China is a country with a complex occurrence of tephritid fruit flies, but at present, most of the domestic research on interspecific competition is on biological behavior; for example, comparing the oviposition selectivity of *B. correcta* and *B. dorsalis* for different hosts. Hosts with high odor selectivity were found to be more suitable for the growth and development of offspring, but a single parameter (development period, survival rate or pupal weight) was not a suitable indicator for host adaptation of tephritids, and it was found that the two species have both overlapping and divergent host ecological niches [137]. After the larval stage, the higher the population density, the more competitive the two species become, although the larvae of *B. correcta* are more resistant to crowding than those of *B. dorsalis*, which showed greater competitive ability. After the pupal stage, the later pupation of mixed pupae may be inhibited by earlier pupation. The asymmetrical mating behavior of both species was reported to have negative effects on the reproduction of the other [137]. Interspecific competition among *B. correcta*, *B. dorsalis* and *Ceratitis capitata* was also explored and showed that there was no mating interference among these three species, but oviposition competition occurred at the appropriate temperature and on guava, and the two *Bactrocera* species had a significant suppressive effect on *C. capitata* [137]. The observation records of the above research can not only be associated with Christensen and Foote’s suggestion that the reason for the absence of *C. capitata* in Southeast Asia may be interspecific competition between *C. capitata* and indigenous flies such as *B. dorsalis* [138] but also serve as a preliminary exploration to answer the question raised by Ma et al., which is similar to the above situation in China. Since 1985, when *C. capitata* was first observed in imported fruits and vegetables in Guangdong Province, it has been identified many times per year in imported products, but there has never been an outbreak in China. It is not known whether this phenomenon is indeed related to the retention role of local tephritid fruit flies; the mechanisms involved are poorly understood, and the precise competitive mechanism is far from clear [139]. In addition to the above research, there are also comparative observations of competition between *Z. cucurbitae* and *Z. tau* regarding oviposit selection preference, the number of offspring in different hosts, etc. [140,141].

## 8. Integrated Management of Tephritid Fruit Flies

### 8.1. Monitoring Surveys

The “attractants + trap” strategy is one of the most effective methods for monitoring surveys and controlling tephritid fruit flies, and commonly used attractants are divided into two main categories: sexual pheromones and food baits, which are placed into efficient and convenient traps. Wu et al., based on the technical bill on tephritid fruit fly monitoring proposed by the International Plant Protection Organization (IPPC), took the opportunity to participate in the international expert group conference to organize the international content related to monitoring techniques and proposed a set of technical element guidelines that can be used by monitoring managers and operators for reference within the boundaries of China; the specific monitoring content was based on “monitoring purposes” (monitoring, detection or delimiting) and “timing and stage” (the period around control or eradication) [142].

### 8.2. Traps

A trap is used to house the attractant devices, and its structure should be suitable for the placement of different traits and to allow maximum effectiveness. At the same time, it should also be easy for the monitoring personnel to hang the traps, maintain them and collect data as well as perform other operations [143]. In ISPM No. 26 (International Standards for Phytosanitary Measures, ISPMs), most of the commonly used traps and the corresponding species of tephritid fruit flies were listed, which were divided into three types; dry types accounted for the majority [144]:

1. Dry type: Cook and Cunningham, ChamP (CH), Jackson, Delta, Lynfield, OBDT, Phase IV, RS, Steiner, ST, YP, Rebell (RB);
2. Wet type: McPhail, Harris;
3. Dry and wet type: Easy trap (ET), MLT, Tephri (TP).

Because of different host species planting or geographical sites, monitoring and control within traditional methods could cost large amounts of human and material resources. These assignments are difficult to carry out, with severe weather conditions or loss of traps, resulting in incorrect data obtained, so new and highly efficient methods are necessary. A monitoring system was designed based on the internet-of-things, which was composed of intelligent fruit fly traps, terminal types of monitoring and remote and mobile types, and the accuracy of the system reached 94.23% [145]. With the development of technology, such as the addition of networks, artificial intelligence and other technologies, digital and intelligent-based fly management systems will become more advanced.

### 8.3. Sex Pheromones

Insect sex pheromones are produced and released by sexually mature individuals, which can induce or provoke the mating of individuals of the same species but the opposite sex [146]. In 2008, China first approved the application of three insect sex pheromones for agricultural pest control, i.e., *B. dorsalis*, *C. capitata* and *Z. cucurbitae*, which started the prelude to the use of insect sex pheromones for pest control [146]. At present, male sexual attractants mainly include volatile components released by flowers in nature, which are used for pollination purposes, such as methyl eugenol (4-allyl-1,2-dimethoxybenzene), raspberry ketone (4-(4-hydroxyphenyl)-2 butanone), cuelure, zingerone and zingerol [147,148,149,150,151,152,153]. However, there are also components of substances volatilized by the insects themselves, e.g., male *C. vesuviana* emit nonanal [154], and undecanol is emitted from the cystic glands of *B. minax* [155].

### 8.4. Food Baits

After eclosion, tephritid fruit flies need to feed on sugar and protein for normal growth and development of eggs and sex organs [111,121]; depending on this property, food source attractants have been developed, such as hydrolyzed protein baits, food source synthetic baits, as well as bacterial fermentation broth and its secondary metabolites.

A laboratory and field comparison between homemade hydrolysates I/II (Fujian Agriculture and Forestry University) and GF-120 (Dow AgroSciences) showed that the homemade hydrolysate I was more effective than GF-120 in attracting females [156]. Subsequently, field measurements in poplar peach and guava orchards revealed that as the concentration of waste brewer’s yeast enzymatic protein and borax increased, the size of the trap increased due to the combination of enzymatic protein and borax, whereas the best concentration for enzymatic protein was 20–25 g/L, and the amount of added borax was 0.06–0.12 mol/L [157].

For waste brewer’s yeast, digestion of *B. dorsalis* was mainly based on its volatiles, so seven main volatiles (3-methyl-1-butanol, benzaldehyde, octyl acetate, phenethyl acetate, ethyl caprylate, benzene acetonitrile and phenethyl alcohol) and their mixtures were screened for their attraction effect. A mixture (200 μL/mL octyl acetate and phenethyl acetate and 100 μL/mL ethyl caprylate) was found to be the most effective, reaching 88.6% [158,159]. In addition, the attraction effect of modified hydrolyzed protein baits in the field showed that the addition of 0.18 g brown sugar to the hydrolyzed protein solution (4.85 g/30 mL) significantly enhanced the attraction effect on *B. dorsalis*, *B. latifrons*, *B. scutellata*, *Z. cucurbitae* and *Z. tau* [160]. On the other hand, aqueous solutions of H-protein bait, GF-120 bait, sugar-vinegar-wine mixture, torula yeast and Jufeng attractant were used in a citrus orchard, and it was found that the H-protein bait was the best, attracting significantly more *B. minax* adults than the sugar–vinegar–wine mixture, torula yeast and Jufeng attractant [161].

### 8.5. Natural Enemy Utilization

Biological control of tephritids is mainly carried out by using natural enemy insects, including predatory and parasitic insects, of which parasitic natural enemies are divided into two categories: parasitic microorganisms and parasitic wasps [162]. Parasitic wasps are the most common method.

Currently, China has introduced stable, large-scale rearing and better natural control of the parasitic wasps *Diachasmimorpha longicaudata* [163] and *Fopius arisanus* [164]. In 2020, *D. longicaudata* was released in guava and poppy orchards in Chongzuo (Guangxi) at a ratio of 1:10 between female wasps and second to third instar larvae of *B. dorsalis*, and its parasitism rate increased rapidly with increasing release amount, indicating that the wasp has a cumulative effect in the field, and its parasitism rate could be as high as 5.97% after three consecutive releases [165]. *Fopius arisanus* was introduced from the United States by Fujian Agriculture and Forestry University in 2005, and a stable experimental population was established [166]. Subsequently, in 2015, a release trial was conducted in the field, and the parasitism rate reached approximately 57% with an effective control time (approximately 15 days); the ratio of *F. arisanus* to *B. dorsalis* was 3:1 [167].

### 8.6. Key Points of the Integrated Management System

It is impractical to rely on one method alone to manage and control tephritid fruit flies, and the control of these flies is a complex system project. Taking *B. dorsalis* as an example, through several years of study, the research team of Professor Zeng Ling from South China Agricultural University proposed the control strategy “agricultural measures as the basis, trapping and control as the main means, and chemical control as the emergency”, and the integrated management project includes the following:

1. “Agricultural measures as the basis”

Monitoring is always an information source as a basis for the work to be carried out. Focus on field sanitation, with the use of physical control methods, because dropped fruit cleaning, bagging efforts, irrigation dips and cutting off the host chain are all fundamental to suppressing the insect population base [168].

2. “Trapping and control as the main means”

Using sexual pheromones in the progress of control as early as possible, such as the unripe fruit stage, could help to reduce subsequent control costs. Long-term and continuous trapping with food and low-toxicity baits, because tephritid flies are mainly infested with larvae in the host and are difficult to kill directly with pharmaceuticals.

3. “Chemical control as the emergency”

In case of emergency or major outbreaks, using 80% Trichlorfon (1000 times) with 150 g brown sugar per week, and a total of three times, the control effect could reach 89.3% [169]. Using 20% Triazophos (500 times) or 1.8% Aifudin (3000–4000 times) per week, and a total of five times, the control effect could be up to 88.9% [168].

Special attention should also be paid to the fact that some flies mentioned in this paper have a wide variety of hosts, and the harvest periods of various hosts may be different or overlap, which is highly conducive for pests to cause damage, so management and control should focus on the harvest period and reduce the damage to below the level of economic damage.

### 8.7. Sterile Insect Technique (SIT)

Sterile insect technology (SIT) is an important component of an integrated pest management system (IPM). SIT in its broadest sense includes irradiation, chemistry, *Wolbachia* symbiosis induction, genetic modification, and combinations of these strategies [170,171,172,173,174]. The most popular and established practice in China is the application of irradiation technology for control, mostly in the case of the tephritid *Bactrocera* species.

In a large-scale experiment, the Institute of Atomic Energy Utilization, Chinese Academy of Agricultural Sciences, in Huishui County, Guizhou Province, released 56,272 and 95,320 radiation-sterile *B. minax* flies in the Zhonglian Orange orchard (500 mu) in 1987 and 1989, respectively, with release ratios of 12.5:1 and 45:1, which reduced the infestation rate from 5 to 8% in normal years to 0.005%, with a significant effect [175]. Later, in 1993 and 1994, researchers expanded the release area to approximately 118 hm^2^ in seven orange orchards containing approximately 100,000 Wenzhou honey tangerine trees, releasing approximately 0.6 million and 1 million sterile flies, which reduced the infestation rate from 5.194% in the three years before to 0.134% in 1993 and 0.098% in 1994 [176]. In 2008, irradiated males of *B. dorsalis* were released in fields three times in Fujian, and the longest dispersal distance of sterile *B. dorsalis* was 207.89 m on the sixth day, and the longest survival period was 15 days. The egg sterility rate of the test group was 91.15% after mating with wild females, but that of the control group was only 21.02%, which indicated a better release effect [177].

## 9. Conclusions

According to the review, the wide range of distribution, broad host range and long adult reproductive life are the main reasons why most tephritid fruit flies cause serious damage, and there have been many attempts in integrated pest management according to the damage characteristics. These attempts have laid a good foundation for improving or discovering new means of control and management. However, we need to be clear at all times in the process. Effective means of control need not simply stack different types of methods but need to be adapted to local conditions, and then, according to the regularity of pest occurrence, not just use chemical agents, especially broad-spectrum insecticides, to avoid the “3R” problem, that is, the resistance, resurgence and residue of pesticides [178], which even affects the safety of humans and livestock.

Subsequent studies can be applied based on the biological properties and environmental regularity in the review; for example, studying the mechanism of phytophagous insects’ olfactory perception of host plant volatiles can help reveal the synergistic evolutionary relationship between phytophagous insects and host plants, screen resistant plant species and develop green pest control technologies [179]. In addition, among the parasitic natural enemies of tephritids, microbial natural enemies include symbiotic bacteria, microsporidian pathogens, fungi, parasitic nematodes and bacteria [162], which are also worth exploring in depth.

## Figures and Tables

**Table 1:** Distribution of tephritid fruit flies (Diptera: Tephritidae) in China (based on the provincial level).

Specific Name	Regions	Provinces	Native Range	First Reported	References
*Bactrocera correcta*	East	Taiwan	India and South-East Asia	1982, Yunnan	[10,11,12,13,14,15,16,17]
	South	Guangxi Zhuangzu Zizhiqu			
	Southwest	Sichuan (only detected in Miyi County) and Yunnan			
*Bactrocera dorsalis*	Central	Hubei and Hunan	South-East China	1911, Taiwan	[16,18,19,20,21,22,23,24,25,26,27,28,29,30,31,32]
	East	Anhui, Jiangsu, Zhejiang, Shanghai Shi ^⊙^, Jiangxi, Fujian and Taiwan			
	South	Guangdong, Guangxi Zhuangzu Zizhiqu, Hainan and Hong Kong ^●^			
	Southwest	Guizhou, Sichuan, Chongqing Shi ^⊙^ and Yunnan			
*Bactrocera latifrons* *(only* *captured by* *bait traps)*	East	Fujian and Taiwan	South-East Asia	-	[16,33,34,35,36,37,38,39,40,41,42,43]
	South	Hainan, Guangdong and Guangxi Zhuangzu Zizhiqu			
	Southwest	Guizhou and Yunnan			
*Bactrocera minax*	Northwest	Shaanxi	China	-	[44,45,46,47,48,49]
	East	Jiangxi and Taiwan			
	Central	Hubei and Hunan			
	South	Guangxi Zhuangzu Zizhiqu			
	Southwest	Guizhou, Sichuan, and Yunnan			
*Bactrocera tsuneonis*	East	Taiwan	East Asia	1940, Sichuan	[16,20,50,51,52,53,54]
	Central	Hunan			
	Southwest	Guizhou, Sichuan, and Yunnan			
*Zeugodacus* *scutellatus* *(only* *captured by* *bait traps)*	North	Shanxi	East Asia	1912, Taiwan	[16,52,55,56,57,58,59,60,61,62,63,64]
	Northwest	Shaanxi (only 6 adults captured by bait traps in 1984)			
	East	Anhui, Jiangxi, Fujian and Taiwan			
	Central	Henan, Hubei and Hunan			
	South	Guangdong, Guangxi Zhuangzu Zizhiqu and Hainan			
	Southwest	Guizhou, Sichuan, Chongqing Shi ^⊙^ and Yunnan			
*Carpomya vesuviana*	Northwest	Xinjiang Uygur Zizhiqu (currently limited in Turpan region and under official control)	India	2007, Xinjiang (Turpan)	[65,66]
*Rhagoletis batava obseuriosa*	North	Nei Mongol Zizhiqu	Russia	1985, Liaoning	[67,68,69]
	Northwest	Shaanxi and Xinjiang Uygur Zizhiqu			
*Zeugodacus cucurbitae*	East	Zhejiang, Jiangxi, Fujian and Taiwan	India	1960, Taiwan	[16,39,70,71,72,73,74,75,76,77,78,79,80]
	Central	Hubei and Hunan			
	South	Guangdong, Guangxi Zhuangzu Zizhiqu, Hainan, and Hong Kong ^●^			
	Southwest	Guizhou, Sichuan Chongqing Shi ^⊙^ and Yunnan			
*Zeugodacus tau*	East	Zhejiang, Jiangxi, Fujian and Taiwan	Asia	1912, Guangdong and Yunnan	[16,59,77,81,82,83,84,85,86,87,88,89]
	Central	Henan, Hubei and Hunan			
	South	Guangdong, Guangxi Zhuangzu Zizhiqu and Hainan			
	Southwest	Guizhou, Sichuan, Chongqing Shi ^⊙^ and Yunnan			

Notes: “⊙” represents municipalities directly under the control of the Central Government, and “●” represents special administrative regions (SAR).

**Table 2:** Records of host plants of tephritid fruit flies in China.

Tephritid Species	Plant Type	Plant Family	Plant Species	Degree of Damage	References
*Bactrocera* *correcta*	Fruit	Anacardiaceae	*Anacardium occidentale*	nd	[3,94,95]
			*Mangifera indica*	+++	
		Annonaceae	*Annona squamosa*	++	
		Combretaceae	*Terminalia catappa*	nd	
		Musaceae	*Musa nana*	++	
		Myrtaceae	*Psidium guajava*	+++	
			*Syzygium samarangense*	nd	
		Oxalidaceae	*Averrhoa carambola*	+++	
		Rhamnaceae	*Ziziphus jujuba*	nd	
			*Ziziphus mauritiana*	++	
		Rosaceae	*Prunus salicina*	+	
			*Prunus* spp.	nd	
			*Pseudocydonia sinensis*	++	
			*Pyrus pyrifolia*	+	
		Rutaceae	*Citrus maxima*	+	
			*Citrus reticulata*	++	
			*Citrus sinensis*	+	
		Sapotaceae	*Manilkara zapota*	nd	
	Vegetable	Cucurbitaceae	*Cucumis sativus*	+	
			*Momordica charantia*	++	
		Solanaceae	*Capsicum annuum*	+	
			*Solanum lycopersicum*	+	
			*Solanum melongena*	+	
*Bactrocera* *dorsalis*	Fruit	Actinidiaceae	*Actinidia fulvicoma*	+	[96,97,98]
		Anacardiaceae	*Mangifera indica*	+/++++	
		Annonaceae	*Desmos chinensis*	+	
		Ebenaceae	*Diospyros kaki*	+	
			*Diospyros morrisiana*	++	
			*Diospyros tutcheri*	+	
		Euphorbiaceae	*Phyllanthus emblica*	+	
		Melastomataceae	*Melastoma dodecandrum*	+	
		Moraceae	*Broussonetia kaempferi*	+	
			*Broussonetia papyrifera*	+	
			*Ficus hirta*	+	
			*Ficus sagittata*	+	
		Musaceae	*Musa nana*	nd	
		Myricaceae	*Myrica rubra*	++	
		Myrtaceae	*Acmena acuminatissima*	+	
			*Cleistocalyx operculatus*	++	
			*Psidium guajava*	+++/++++	
			*Rhodomyrtus tomentosa*	++	
			*Syzygium jambos*	++++	
			*Syzygium levinei*	+	
			Syzygium samarangense	++++	
		Oxalidaceae	*Averrhoa carambola*	+++	
		Punicaceae	*Punica granatum*	+++	
		Rhamnaceae	*Ziziphus jujuba*	++++	
			*Ziziphus spp.*	nd	
		Rhizophoraceae	*Carallia brachiata*	++	
		Rosaceae	*Amygdalus davidiana*	++	
			*Duchesnea indica*	+	
			*Eriobotrya fragrans*	+	
			*Eriobotrya japonica*	++/++++	
			*Malus pumila*	+	
			*Prunus mume*	+	
			*Prunus persica*	+/++++	
			*Prunus phaeosticta*	+	
			*Prunus salicina*	+	
			*Pseudocydonia sinensis*	+	
			*Pyrus calleryana*	+	
			*Pyrus pyrifolia*	+	
			*Rubus leucanthus*	+	
			*Rubus reflexus*	+	
			*Rubus rosifolius*	+	
			*Rubus sumatranus*	+	
		Rutaceae	*Citrus limon*	+	
			*Citrus maxima*	+	
			*Citrus reticulata*	+++	
			*Clausena lansium*	++	
			*Fortunella hindsii*	++	
		Sapotaceae	*Manilkara zapota*	+	
		Vitaceae	*Cayratia japonica*	+	
			*Vitis amurensis*	+	
			*Vitis vinifera*	+	
	Vegetable	Cucurbitaceae	*Cucumis melo*	+	
			*Cucumis sativus*	++	
			*Cucurbita moschata*	+	
			*Luffa aegyptiaca*	++++	
			*Momordica charantia*	+	
			*Sechium edule*	+	
		Solanaceae	*Capsicum annuum*	+	
			*Solanum lycopersicum*	++	
			*Solanum melongena*	+	
*Bactrocera latifrons*	Vegetable	Solanaceae	*Capsicum annuum*	+	[33,99,100]
			*Solanum melongena*	+	
*Bactrocera* *minax*	Fruit	Rutaceae	*Citrus aurantium*	nd	[47]
			*Citrus erythrosa*	nd	
			*Citrus junos*	nd	
			*Citrus limon*	nd	
			*Citrus maxima*	+++/++++	
			*Citrus medica*	+++/++++	
			*Citrus paradisi*	nd	
			*Citrus poonensis*	+/++++	
			*Citrus reticulata*	nd	
			*Citrus sinensis*	+/++/+++/++++	
			*Citrus tangerina*	+/+++	
			*Citrus unshiu*	+/++/+++/++++	
			*Fortunella margarita*	nd	
			*Poncirus trifoliata*	nd	
*Bactrocera* *tsuneonis*	Fruit	Rutaceae	*Citrus aurantium*	nd	[101,102]
			*Citrus reticulata*	nd	
			*Citrus sinensis*	nd	
			*Fortunella japonica*	nd	
*Carpomya* *vesuviana*	Fruit	Rhamnaceae	*Ziziphus* spp.	nd	[65]
*Rhagoletis* *batava* *obseuriosa*	Fruit	Elaeagnaceae	*Hippophae* spp.	nd	[68]
*Zeugodacus* *cucurbitae*	Vegetable	Cucurbitaceae	*Benincasa hispida*	nd	[97,103]
			*Citrullus lanatus*	nd	
			*Cucumis sativus*	++++	
			*Cucurbita moschata*	nd	
			*Cucurbita pepo*	nd	
			*Luffa aegyptiaca*	++++	
			*Momordica charantia*	++	
			*Sechium edule*	++	
*Zeugodacus scutellatus*	Vegetable	Cucurbitaceae	Cucurbitaceae flowers	nd	[16,104]
*Zeugodacus* *tau*	Vegetable	Cucurbitaceae	*Benincasa hispida*	nd	[97,105,106]
			*Citrullus lanatus*	++	
			*Cucumis sativus*	+/++	
			*Cucurbita moschata*	++/+++/++++	
			*Cucurbita pepo*	nd	
			*Luffa aegyptiaca*	+/++	
			*Momordica charantia*	+	
			*Sechium edule*	++	

Notes: “+” represents the degree of damage (<10%: +, 10–30%: ++, 30–50%: +++, >50%: ++++); “nd” represents there is no record of the degree of harm although there is a host; “p” represents possible hosts.

## Data Availability

No new data were created or analyzed in this study. Data sharing is not applicable to this article.
