# Correction: He et al. Biology, Ecology and Management of Tephritid Fruit Flies in China: A Review. *Insects* 2023, *14*, 196

**DOI:** 10.3390/insects15020093

**Published:** 2024-01-31

**Authors:** Yuxin He, Yijuan Xu, Xiao Chen

**Affiliations:** 1Guangdong Laboratory for Lingnan Modern Agriculture, Department of Entomology, South China Agricultural University, Guangzhou 510642, China; 2Henry Fok School of Biology and Agriculture, Shaoguan University, Shaoguan 512005, China

## Error in Figure/Table

In the original publication [1], there was a mistake in Figure 1 and Table 1 and Table 2 as published. Overall, the issue that needs to be corrected relates to the fact that the distribution range of tephritid fruit flies is not as large as previously described. Although they were mentioned in the literature, sometimes there is no evidence of the field collection of adults or larvae. In other instances, the fruit can only be infested when the hard peel is mechanically damaged and therefore cannot be considered a host.

The corrected Figure 1 and Table 1 and Table 2 are shown below.

**Figure 1 insects-15-00093-f001:**
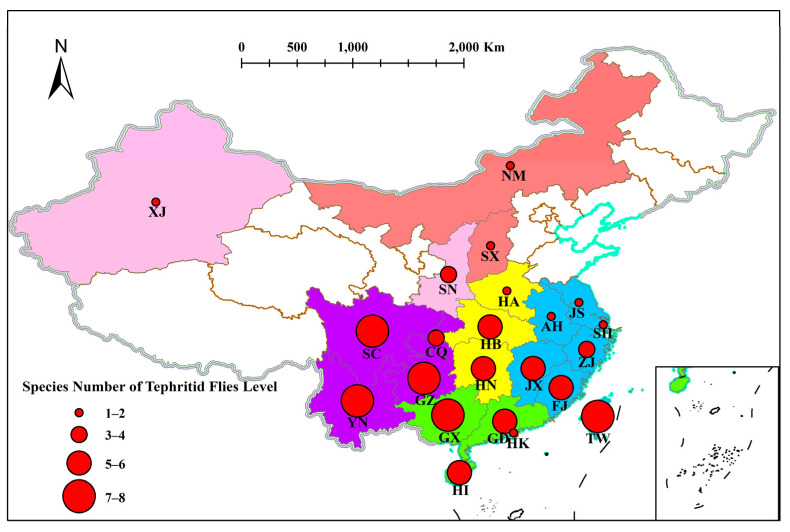
Distribution of tephritid fruit flies in each province of China. The size of the solid circles in the figure represents the species number of tephritid fruit flies. Colors in the figure represent different regions (Red, yellow, green, blue, purple, and pink represent North, Central, South, East, Southwest, and Northwest China respectively). Abbreviations: AH, Anhui; CQ, Chongqing Shi; FJ, Fujian; GD, Guangdong; GX, Guangxi Zhuangzu Zizhiqu; GZ, Guizhou; HA, Henan; HB, Hubei; HI, Hainan; HK, Hong Kong; HN, Hunan; JS, Jiangsu; JX Jiangxi; NM, Nei Mongol Zizhiqu; SC, Sichuan; SH, Shanghai Shi; SN, Shaanxi, SX, Shanxi; XJ Xinjiang Uygur Zizhiqu, TW, Taiwan; YN, Yunnan; ZJ, Zhejiang. The map data was generated by Geospatial Data Cloud (https://www.gscloud.cn, accessed on 4 February 2022) and Alibaba Cloud (DataV.GeoAtlas, http://datav.aliyun.com/portal/school/atlas/area_selector, accessed on 4 February 2022). The spa-tial analysis function was via ArcGIS (version 10.7) and Mapshaper (https://mapshaper.org, accessed on 4 February 2022).

**Table 1 insects-15-00093-t001:** Distribution of tephritid fruit flies (Diptera: Tephritidae) in China (based on the provincial level).

Specific Name	Regions	Provinces	Native Range	First Reported	References
*Bactrocera correcta*	East	Taiwan	India and South-East Asia	1982, Yunnan	[10–17]
	South	Guangxi Zhuangzu Zizhiqu			
	Southwest	Sichuan (only detected in Miyi County) and Yunnan			
*Bactrocera dorsalis*	Central	Hubei and Hunan	South-East China	1911, Taiwan	[16,18–32]
	East	Anhui, Jiangsu, Zhejiang, Shanghai Shi ^⊙^, Jiangxi, Fujian and Taiwan			
	South	Guangdong, Guangxi Zhuangzu Zizhiqu, Hainan and Hong Kong ^●^			
	Southwest	Guizhou, Sichuan, Chongqing Shi ^⊙^ and Yunnan			
*Bactrocera latifrons (only captured by bait traps)*	East	Fujian and Taiwan	South-East Asia	-	[16,33–43]
	South	Hainan, Guangdong and Guangxi Zhuangzu Zizhiqu			
	Southwest	Guizhou and Yunnan			
*Bactrocera minax*	Northwest	Shaanxi	China	-	[44–49]
	East	Jiangxi and Taiwan			
	Central	Hubei and Hunan			
	South	Guangxi Zhuangzu Zizhiqu			
	Southwest	Guizhou, Sichuan, and Yunnan			
*Bactrocera tsuneonis*	East	Taiwan	East Asia	1940, Sichuan	[16,20,50–54]
	Central	Hunan			
	Southwest	Guizhou, Sichuan, and Yunnan			
*Zeugodacus scutellatus (only captured by bait traps)*	North	Shanxi	East Asia	1912, Taiwan	[16,52,55–64]
	Northwest	Shaanxi (only 6 adults captured by bait traps in 1984)			
	East	Anhui, Jiangxi, Fujian and Taiwan			
	Central	Henan, Hubei and Hunan			
	South	Guangdong, Guangxi Zhuangzu Zizhiqu and Hainan			
	Southwest	Guizhou, Sichuan, Chongqing Shi ^⊙^ and Yunnan			
*Carpomya vesuviana*	Northwest	Xinjiang Uygur Zizhiqu (currently limited in Turpan region and under official control)	India	2007, Xinjiang (Turpan)	[65,66]
*Rhagoletis batava obseuriosa*	North	Nei Mongol Zizhiqu	Russia	1985, Liaoning	[67–69]
	Northwest	Shaanxi and Xinjiang Uygur Zizhiqu			
*Zeugodacus cucurbitae*	East	Zhejiang, Jiangxi, Fujian and Taiwan	India	1960, Taiwan	[16,39,70–80]
	Central	Hubei and Hunan			
	South	Guangdong, Guangxi Zhuangzu Zizhiqu, Hainan and Hong Kong ^●^			
	Southwest	Guizhou, Sichuan Chongqing Shi ^⊙^ and Yunnan			
*Zeugodacus tau*	East	Zhejiang, Jiangxi, Fujian and Taiwan	Asia	1912, Guangdong and Yunnan	[16,59,77,81–89]
	Central	Henan, Hubei, and Hunan			
	South	Guangdong, Guangxi Zhuangzu Zizhiqu and Hainan			
	Southwest	Guizhou, Sichuan, Chongqing Shi ^⊙^ and Yunnan			

Notes: “⊙” represents municipalities directly under the control of the Central Government, and “●” represents special administrative regions (SAR).

**Table 2 insects-15-00093-t002:** Records of host plants of tephritid fruit flies in China.

Tephritid Species	Plant Type	Plant Family	Plant Species	Degree of Damage	References
*Bactrocera* *correcta*	Fruit	Anacardiaceae	*Anacardium occidentale*	nd	[3,94,95]
			*Mangifera indica*	+++	
		Annonaceae	*Annona squamosa*	++	
		Combretaceae	*Terminalia catappa*	nd	
		Musaceae	*Musa nana*	++	
		Myrtaceae	*Psidium guajava*	+++	
			*Syzygium samarangense*	nd	
		Oxalidaceae	*Averrhoa carambola*	+++	
		Rhamnaceae	*Ziziphus jujuba*	nd	
			*Ziziphus mauritiana*	++	
		Rosaceae	*Prunus salicina*	+	
			*Prunus* spp.	nd	
			*Pseudocydonia sinensis*	++	
			*Pyrus pyrifolia*	+	
		Rutaceae	*Citrus maxima*	+	
			*Citrus reticulata*	++	
			*Citrus sinensis*	+	
		Sapotaceae	*Manilkara zapota*	nd	
	Vegetable	Cucurbitaceae	*Cucumis sativus*	+	
			*Momordica charantia*	++	
		Solanaceae	*Capsicum annuum*	+	
			*Solanum lycopersicum*	+	
			*Solanum melongena*	+	
*Bactrocera* *dorsalis*	Fruit	Actinidiaceae	*Actinidia fulvicoma*	+	[96–98]
		Anacardiaceae	*Mangifera indica*	+/++++	
		Annonaceae	*Desmos chinensis*	+	
		Ebenaceae	*Diospyros kaki*	+	
			*Diospyros morrisiana*	++	
			*Diospyros tutcheri*	+	
		Euphorbiaceae	*Phyllanthus emblica*	+	
		Melastomataceae	*Melastoma dodecandrum*	+	
		Moraceae	*Broussonetia kaempferi*	+	
			*Broussonetia papyrifera*	+	
			*Ficus hirta*	+	
			*Ficus sagittata*	+	
		Musaceae	*Musa nana*	nd	
		Myricaceae	*Myrica rubra*	++	
		Myrtaceae	*Acmena acuminatissima*	+	
			*Cleistocalyx operculatus*	++	
			*Psidium guajava*	+++/++++	
			*Rhodomyrtus tomentosa*	++	
			*Syzygium jambos*	++++	
			*Syzygium levinei*	+	
			*Syzygium samarangense*	++++	
		Oxalidaceae	*Averrhoa carambola*	+++	
		Punicaceae	*Punica granatum*	+++	
		Rhamnaceae	*Ziziphus jujuba*	++++	
			*Ziziphus spp.*	nd	
		Rhizophoraceae	*Carallia brachiata*	++	
		Rosaceae	*Amygdalus davidiana*	++	
			*Duchesnea indica*	+	
			*Eriobotrya fragrans*	+	
			*Eriobotrya japonica*	++/++++	
			*Malus pumila*	+	
			*Prunus mume*	+	
			*Prunus persica*	+/++++	
			*Prunus phaeosticta*	+	
			*Prunus salicina*	+	
			*Pseudocydonia sinensis*	+	
			*Pyrus calleryana*	+	
			*Pyrus pyrifolia*	+	
			*Rubus leucanthus*	+	
			*Rubus reflexus*	+	
			*Rubus rosifolius*	+	
			*Rubus sumatranus*	+	
		Rutaceae	*Citrus limon*	+	
			*Citrus maxima*	+	
			*Citrus reticulata*	+++	
			*Clausena lansium*	++	
			*Fortunella hindsii*	++	
		Sapotaceae	*Manilkara zapota*	+	
		Vitaceae	*Cayratia japonica*	+	
			*Vitis amurensis*	+	
			*Vitis vinifera*	+	
	Vegetable	Cucurbitaceae	*Cucumis melo*	+	
			*Cucumis sativus*	++	
			*Cucurbita moschata*	+	
			*Luffa aegyptiaca*	++++	
			*Momordica charantia*	+	
			*Sechium edule*	+	
		Solanaceae	*Capsicum annuum*	+	
			*Solanum lycopersicum*	++	
			*Solanum melongena*	+	
*Bactrocera latifrons*	Vegetable	Solanaceae	*Capsicum annuum*	+	[33,99,100]
			*Solanum melongena*	+	
*Bactrocera* *minax*	Fruit	Rutaceae	*Citrus aurantium*	nd	[47]
			*Citrus erythrosa*	nd	
			*Citrus junos*	nd	
			*Citrus limon*	nd	
			*Citrus maxima*	+++/++++	
			*Citrus medica*	+++/++++	
			*Citrus paradisi*	nd	
			*Citrus poonensis*	+/++++	
			*Citrus reticulata*	nd	
			*Citrus sinensis*	+/++/+++/++++	
			*Citrus tangerina*	+/+++	
			*Citrus unshiu*	+/++/+++/++++	
			*Fortunella margarita*	nd	
			*Poncirus trifoliata*	nd	
*Bactrocera* *tsuneonis*	Fruit	Rutaceae	*Citrus aurantium*	nd	[101,102]
			*Citrus reticulata*	nd	
			*Citrus sinensis*	nd	
			*Fortunella japonica*	nd	
*Carpomya* *vesuviana*	Fruit	Rhamnaceae	*Ziziphus* spp.	nd	[65]
*Rhagoletis* *batava* *obseuriosa*	Fruit	Elaeagnaceae	*Hippophae* spp.	nd	[68]
*Zeugodacus* *cucurbitae*	Vegetable	Cucurbitaceae	*Benincasa hispida*	nd	[97,103]
			*Citrullus lanatus*	nd	
			*Cucumis sativus*	++++	
			*Cucurbita moschata*	nd	
			*Cucurbita pepo*	nd	
			*Luffa aegyptiaca*	++++	
			*Momordica charantia*	++	
			*Sechium edule*	++	
*Zeugodacus* *scutellatus*	Vegetable	Cucurbitaceae	Cucurbitaceae flowers	nd	[16,104]
*Zeugodacus* *tau*	Vegetable	Cucurbitaceae	*Benincasa hispida*	nd	[97,105,106]
			*Citrullus lanatus*	++	
			*Cucumis sativus*	+/++	
			*Cucurbita moschata*	++/+++/++++	
			*Cucurbita pepo*	nd	
			*Luffa aegyptiaca*	+/++	
			*Momordica charantia*	+	
			*Sechium edule*	++	

Notes: “+” represents the degree of damage (<10%: +, 10–30%: ++, 30–50%: +++, >50%: ++++); “nd” represents there is no record of the degree of harm although there is a host; “p” represents possible hosts.

## Missing Citation

In the original publication, “Refs. [10–12,31–32,44,81]” was not cited. The citation has now been inserted in Table 1. 

In the original publication, “Ref. [104]” was not cited. The citation has now been inserted in Table 2. 

## Text Correction

There was an error in the original publication: *Bactrocera scutellata* was referred to instead of *Zeugodacus scutellatus*. *Bactrocera scutellata* has been changed to *Zeugodacus scutellatus* due to a recent classification revision.

A correction has been made to the second paragraph in the Introduction section, Table 1 and Table 2.

“…which are the most studied in China, especially *Bactrocera correcta* (Bezzi), *Bactrocera dorsalis* (Hendel), *Bactrocera latifrons* (Hendel), *Bactrocera minax* (Enderlein), *Bactrocera tsuneonis* (Miyake), *Carpomya vesuviana* (Costa), *Rhagoletis batava obseuriosa* (Kolomiets), *Zeugodacus cucurbitae* (Coquillett), *Zeugodacus scutellatus* (Hendel) and *Zeugodacus tau* (Walker).”

A correction has been made to Section 2.2 Distribution. We rephrased the first sentence to “The data presented in Figure 1 and Table 1 were obtained from literature reports with the field evidence. Fujian and Taiwan Provinces in East China, Guangdong Province and the Guangxi Zhuang Zizhiqu in South China, and Guizhou, Sichuan and Yunnan Provinces in Southwest China are the areas where the tephritid fruit flies overlap many times.…”

## References

Due to the change in the distribution range of some species (showed in Table 1), the following references are no longer necessary for citation in the updated manuscript.[3] White, I.M.; Elson-Harris, M.M. *Fruit Flies of Economic Significance: Their Identification and Bionomics*, 1st ed.; Oxford University Press: Oxford, UK, 1992; ISBN 978-0-85198-790-3.[10] Database of Major Invasive Animal Species. Available online: http://museum.ioz.ac.cn/iad/View/Site/Species.aspx (accessed on 4 February 2023).[21] Gong, Q.T.; Zhang, K.P.; Li, S.H.; Jia, H.Z.; Wu, H.B.; Sun, R.H. Occurrence, damage, prevention and control of *Bactrocera dorsalis* (Hendel). *Deciduous Fruits* **2022**, *54*, 49–52. https://doi.org/10.13855/j.cnki.lygs.2022.01.015.[37] Chen, P.; Ye, H. Fruit fly diversity analysis at five regions in the western Yunnan, China. *Acta Ecol. Sin.*
**2009**, *29*, 2953–2961.[49] Gong, X.Z.; Chen, W.H.; Bai, Z.L.; Gan, X.J.; Liao, Y.M. Effects of attractants on the trapping of Bactrocera (Tetradacus) tsuneonis (Miyake). *Plant Quar*. **2008**, *22*, 285–287. https://doi.org/10.3969/j.issn.1005-2755.2008.05.005.[63] Zhang, X.Y.; Chen, G.Q.; Meng, Y.Q.; Huang, Z.D. Population dynamics and effect evaluation of sexual pheromones for monitoring tephritids flies in orange orchards with mixed planting of fruits and vegetables in Taizhou. *J. Zhejiang Agric. Sci.* **2011**, 1368–1370. https://doi.org/10.16178/j.issn.0528-9017.2011.06.040.[69] Ge, B.W.; Li, G.H.; Zhang, Y.A.; Fan, Y.F. Preliminary study of Rhagoletis batava obseuriosa. *Liaoning For. Sci. Technol.* **1988**, 45–46+61. Available online: https://oversea.cnki.net/kns/manage/export?filename=lnlk198803015&dbname=CJFD8589 (accessed on 2 January 2023).[70] Chen, X.D.; Dang, X.D.; Li, F. Characteristics and analysis of the zone system of hippophae insects in Shaanxi. *Hippophae* **2001**, *14*, 23–26.[71] Liu, J.J. Major pests and weeds of hippophae in Heilongjiang Province and integrated control. *Hippophae* **2005**, *18*, 11–12.[90] Yang, Y.L.; Wu, S.A.; Zheng, M.H. Initial reports of *Zeugodacus tau* in Shanxi Province. *Plant Quar*. **1994**, *8*, 330–331.[93] Ismay, J.W. Fruit Flies of Economic Significance: Their Identification and Bionomics. By Ian M. White and Marlene, M. Elson- Harris. (Wallingford: CAB International, 1991). *Bull. Entomol. Res.*
**1992**, *82*, 433.[132] Zhang, Z.Y.; Zhao, B.; Zhang, L.; Liang, H.J. The host preference experiment of *Bactrocera correcta*. *Chin. J. Appl. Entomol.*
**2011**, *48*, 359–363.

Below references were added in the updated manuscript.
[10] Liu, X.; Zhang, L.; Haack, R.A.; Liu, J.; Ye, H. A noteworthy step on a vast continent: new expansion records of the guava fruit fly, *Bactrocera correcta* (Bezzi, 1916)(Diptera: Tephritidae), in mainland China. *BioInvasions Rec.*
**2019**, *8*, 530–539.[11] Liu, X.; Jin, Y.; Ye, H. Recent spread and climatic ecological niche of the invasive guava fruit fly, *Bactrocera correcta*, in mainland China. *J. Pest Sci.*
**2013**, *86*, 449–458.[12] Yu, J.Y.; Ren, K.L.; Xue, W.P.; Li, T.Q.; Wang, X.J.; Geng, K. The species and population dynamics of Tephritid fruit flies in the macaque peach orchard of Xiuwen County. *S. China Fruits*
**2022**, *51*, 117–121.[31] Aketarawong, N.; Bonizzoni, M.; Thanaphum, S.; Gomulski, L.; Gasperi, G.; Malacrida, A.R.; Gugliemino, C. Inferences on the population structure and colonization process of the invasive oriental fruit fly, *Bactrocera dorsalis* (Hendel). *Mol. Ecol.*
**2007**, *16*, 3522–3532.[32] Wan, X.; Nardi, F.; Zhang, B.; Liu, Y. The oriental fruit fly, *Bactrocera dorsalis*, in China: origin and gradual inland range expansion associated with population growth. *PLoS ONE*
**2011**, *6*, e25238.[44] Cui, Z.; Zhou, Q.; Liu, Y.; Si, P.; Wang, Y. Molecular identification of citrus fruit flies and genetic diversity analysis of *Bactrocera minax* (Diptera: Tephritidae) populations in China based on mtDNA COI gene sequences. *Acta Entomol. Sin.*
**2020**, *63*, 85–96.[81] Wang, Y.T.; Bai, Q.; Chen, H.S.; Tian, Z.Y.; Gao, X.Y.; Zhou, Z.S. Distribution differences between *Zeugodacus cucurbitae* and *Zeugodacus tau* in China. *J. Environ. Entomol.*
**2022**, *44*, 1170–1175.[104] Al Baki, M.A.; Keum, E.; Kim, H.; Song, Y.; Kim, Y.; Kwon, G.; Park, Y. Age grading and gene flow of overwintered *Bactrocera scutellata* populations. *J. Asia-Pac. Entomol.*
**2017**, *20*, 1402–1409.

Due to these corrections, the order of some references has been adjusted accordingly. The order of some other references needs to be adjusted, please see details as below:

Citation in the original publicationNew citation in updated publication[93][3][46][47][45][48][48][53][64][57][65][58][55][63][60][64][68][68][80][70][84][71][79][72][76][73][75][74][78][75][53][82][89][84][85][89][99][94][51][102][153][149][155][151]

The authors state that the scientific conclusions are unaffected. This correction was approved by the Academic Editor. The original publication has also been updated.
